# Finding New Components of the Mammalian Immune System*

**DOI:** 10.5041/RMMJ.10245

**Published:** 2016-07-28

**Authors:** Bruce Beutler

**Affiliations:** Nobe Prize Laureate in Physiology or Medicine, 2011. From the Center for Genetics of Host Defense, UT Southwestern Medical Center, Dallas, TX, USA

**Keywords:** Forward genetics, mammalian biology, NOMID

## Abstract

The use of forward genetics to analyze mammalian biology has been dramatically accelerated by methods that make it possible instantly to determine which mutation causes a phenotype. Now it is possible to discover gene function as rapidly as mutations can be created and screened: approximately 1,000 coding changes per week are interrogated in our laboratory. Moreover, it is possible to know approximately how much damage has been done to the genome over time. We estimate that we have damaged or destroyed about one-quarter of all protein encoding genes and tested the effects of variant alleles within these genes three times or more in a set of phenotypic assays that interest us. Only about two years were required to reach this level of saturation.

## INTRODUCTION

Thank you all of you for the very kind remarks made about my father and mother. I can tell you they would be very proud of the program they started—my father would be delighted if he were still around to see it. One of my favorite quotes from my father was that the “measure of a scientist isn’t in the size of his laboratory but in the size of his ideas.”

I think that applies here. He would be proud of the portfolio that has been started. The amount of money that was donated was, after all, not enormous, but I think it can light a fire. Like me, he was the type who liked to learn of progress in diverse areas of science. I think he got rather bored by focusing just on one small area of science—he was too intellectually restless for that.

I would like to give you an update on what I talked about some time ago.

## THE PATH TO DISCOVERY

We think of biological organisms as machines of a kind and would like to know how they work, just as we might reverse-engineer a car or a radio or almost any kind of machine of human construction. The first step would be knowing all of the parts. The way that biologists traditionally create a list of parts needed for a biological machine to operate is forward genetics. In other words, we randomly damage the genome of an organism again and again. We screen for exceptional animals that have a problem with whatever process we are interested in, and then we track down the mutation and understand at last something that we did not understand before.

Forward genetics was traditionally practiced in bacteriophage and bacteria, then in worm and in fruitflies. It took a fairly heroic effort when it came to mammals. In the early days of mouse mutagenesis, which was practiced even several decades ago, a biologist could expect to spend years tracking down a causative mutation if a phenotype was observed. The genetic route to finding the mutation always proceeded through phases of genetic mapping, physical mapping, and then gene identification, since, for most parts of the genome, gene content was a mystery. Finally, one would treat the genes as candidates and look for a mutation that made the difference between a reference strain and the mutant strain.

This was a blind process when it began. The only way we knew that something was being accomplished when we mutagenized was to observe excessive numbers of unusual-looking mice. We knew we were having a phenotypic effect because of them, but we didn’t know how much mutation we were introducing into the progenitor of a given pedigree. There was wild guesswork about that. Some said the rate of mutation was one in 100,000 bases, some said one in a million—nobody really knew. Even intensive single-locus testing, in which a null allele was used to “expose” damage to a normal allele, gave only vague estimates of what was going on.

There were some technological advances that accelerated the process, beginning in the early 2000s. First, in 2002, the mouse genome sequence was published in an annotated form. Then it was no longer necessary to perform physical mapping. Genetic mapping was enough, and when one had established a critical region one had a list of candidate genes. It was then much easier to find a mutation. Still, it could be challenging. There might be a critical region with 30, 40, 50 genes in it, each with seven or eight exons on average. At some point we developed a robotic means of covering a critical region. We would make a computer drive a robot to prepare sequencing reactions from a pre-computed set of primers, and we would go from one end of the critical region to the other, looking at all of the coding sequence. We knew from experience that if we saw a phenotype, it almost always came from a change in coding sequence.

By 2009, after much anticipation, we knew that we could sequence the whole genome of the mouse in an ordinary laboratory. This cost $20,000 at the time, but that seemed like a bargain if one could see every mutation. If we had a recessive phenotype, we would generally take this approach to find it. We would lightly cover the genome to about three X depth, find the majority of mutations, and usually there was only a single mutation within the established critical region. That was almost invariably the causative mutation.

Progress was further accelerated with the advent of whole-exome sequencing. We could go into great depth and even expect to find heterozygous mutations. This opened the possibility of knowing all of the mutations in a pedigree up front, before the G3 mice had even been produced by inbreeding. Male mice born to a mutagen-treated male could be sequenced. Then we knew how many mutations were brought to each animal from the sperm of the mutagenized father. We’ve been at this for a while now and covered several thousand exomes. I can tell you the average number of coding changes transmitted from a mutagenized male is 63—the modal number is somewhere between 70 and 75, and there is fairly wide variance in the curve. We are looking here at the number of mutations per sperm and here at the number of observed instances of that number of mutations. We are looking in total at 1,197 whole-exome sequencing jobs.

Whole-exome sequencing also opened the possibility of archiving mutations in the mouse. By about 2006 or so it had become possible to freeze the sperm of C57BL/6J mice and then regenerate them by intracytoplasmic sperm injection (ICSI). Later techniques got even better, and it was possible to do *in-vitro* fertilization (IVF) to recreate mice. So it made sense to sequence every G1 mouse. In this way we developed an extensive library of mutations.

Beginning in 2011, just a little over four years ago, we began to archive mutations. To this date we have archived 303,039 incidental mutations (so named because, when we began, we thought of most of these mutations as “incidental” to the one that caused phenotype) that change coding sense. These mutations fall into 22,476 genes. In other words, we have altered about 90% of all the protein-encoding genes in the mouse by mutation.

A total of 24,728 of these mutations are putative null alleles. The null alleles are falling into 45.6% of all protein-encoding genes. So we have managed with chemical mutagenesis to knock out nearly half of all the genes in the mouse. About 113,000 mutations are either null or judged by PolyPhen-2, a predictive software program that looks at the likelihood of damage to a protein, to be “probably damaging.” These alleles fall into 76% of all protein-encoding genes.

We tend to take the percentage of genes with null alleles as an underestimate of how much damage we’ve done to the genome. It is a rule of thumb that if one sees a phenotype it is half as often caused by a null allele as by a missense allele. So the number of genes that have been mutated to putative null ignores much of the damage done by ENU. On the other hand, the percentage of genes altered by “either null or probably damaging alleles” is an overestimate of the damage done—it’s far too liberal. We know that PolyPhen-2 overcalls damage, and we take the exact average of “probably null” and “probably null or probably damaging” as our best estimate of “mutation to phenovariance.”

In this collection of mutations, however, we have no assurance that every mutant allele was brought to homozygosity. Some were and some were not. That for a long time was quite a barrier to progress. A great deal of information was lost because we didn’t have a way effectively to genotype all of the descendants of every G1 mouse, and therefore know precisely which mutations were examined in the homozygous state and how many times they were examined.

The promise of truly transparent forward genetics depended on fast and inexpensive genotyping, so that we would know the genotype of every mouse born, at every mutated locus identified in its grandfather. Furthermore, by 2011, it was clear to us that the rate-limiting step in the whole process of positional cloning was genetic mapping. Postdoctoral associates who did the screening were accumulating many more mutant phenotypes than they could possibly solve. The traditional method of making a homozygous stock, outcrossing it to a marker strain, and then backcrossing it, establishing a critical region, and tracking down the gene was untenable because we were accumulating a huge backlog of mutants to analyze.

Beginning in February of 2014 we developed a revolutionary method for measuring cumulative damage to the genome while simultaneously maintaining surveillance over the function of all genes at a phenotypic level. This allowed us to map and positionally to clone in real time, finding instantly, more or less, the mutations that caused phenotype.

I want to tell you quickly how this works. Just as always, we mutagenized G0 mice, we bred them to make grandfathers of the pedigree, the G1 males. Those in turn were bred to make daughters (the G2 females). The G1 males were then crossed to their daughters to make G3 mice that might be homozygous for mutations found in the G1. In the new strategy, every G1 mouse was sequenced as soon as it was weaned. If it had more than 30 mutations, we went ahead and bred it. Otherwise we deemed it insufficiently mutagenized to pursue. When G2 and G3 mice were born, before any phenotypic screening was performed, they were genotyped at every locus that mutated in the G1 grandfather (G3 mice) or father (G2 mice).

Using this procedure, we are now able to know whether there is homozygosity for the mutations in the G1 progenitor. This means that we must do about 5 million genotypes per year. So this is quite a major part of the effort. It is done by Ion Torrent sequencing, which is not necessarily high-throughput, but is nimble. One can perform customized sequencing for many G1 mutation sets per week. Then the mice are released for screening as whole pedigrees. The genotypic data are already resident in the computer, awaiting phenotypic data. As soon as phenotypic data are uploaded to the computer, a search for correlation between phenotype and genotype is triggered, using recessive, dominant, and additive models of inheritance. The computer immediately informs us whether there is a phenotype, in any of approximately 150 different screens performed. If a phenotype is present, it will tell us the causative mutation. If there is no causative mutation, then we dismiss the phenotype as being an artifact or non-genetic. Finally, when causative mutations are found, they are verified by clustered regularly interspaced short palindromic repeats (CRISPR) technology. Either we recreate the mutation or we knock out the gene if we know that a knockout is viable in the state we wish to study it (homozygous or heterozygous).

This permits our laboratory to assign cause and effect to mutations within about one hour of seeing a phenotype. There is no longer any need for the old-style positional cloning. That applies to all phenotypes studied, whether they are quantitative or qualitative, visible or immunologic, and the cost is independent of the number of phenotypes that emanate from a pedigree. Often there is more than one phenotype per pedigree, given that there are on average 63 mutations causing coding change, and given that we have so many screens. We don’t require 100% penetrance to track down a causative mutation. Low penetrance is a common circumstance in that one observes considerable variance in immune performance. For example, if you look at the antibody response there will often be overlap between affected and unaffected mice. Nonetheless the computer picks out the correct mutation, statistically testing the null hypothesis of non-association between each variant allele and the phenotype that is observed. Furthermore, we can detect and measure how much saturation has been achieved as a screen progresses within defined limits of error, and we can exonerate specific genes of interest. If you happen to read a paper, or if you should happen to write a paper claiming that the knockout of a particular gene has a particular immunological effect, we might compare your data to our own, examining the phenotypic performance of several null alleles of the same gene. In time, when we have covered all of the genome, the phenotypic effects of mutations at all loci will be known.

What screens do we pursue? First we simply look at the mice, then we weigh them. We do a glucose tolerance test. We look at innate immune performance, harvesting the macrophages of each mouse and checking the response to various inducers of an inflammatory response. We look also at adaptive immune performance, giving two T-dependent and one T-independent challenge, and we look for allergies. We look for developmental defects or for activation defects in the blood of the mice by flow cytometry. Then the pipeline is split, and we look for the ability to cope with mouse cytomegalovirus infection on the one hand, or for the ability to repair a subtle injury caused by a low dose of dextran sodium sulphate [DSS in slide at 19:52]. Normally, mice tolerate DSS at the dose administered. But exceptional mice with certain mutations develop severe colitis. Then the mice go on to certain neurobehavioral screens. We try to get the most out of them that we possibly can.

## CURRENT STATUS

As of today, 85,074 point mutations that change coding sense in 18,724 genes have been collected for screening, and almost all of those have been subjected to the screening pipeline in full. These mutations came from a pool of 44,906 G3 mice, examined over a period of two years. These G3 mice belonged to 1,587 pedigrees. In all, the mutations were queried a total of 6.6 million times by the computer. That means, in every mouse, every mutation (of which there might be 60 or so), was checked in 150 different screens, testing to see whether the null hypothesis of non-association in recessive, additive, or dominant models of inheritance could be rejected. This is a fairly computationally intensive process, and it requires a cluster computer for adequate speed.

We take it somewhat arbitrarily that a good test of the effect of a mutation is to look at it in the homozygous state three times or more. Obviously more testing permits the detection of more subtle phenotypic effects. But a robust phenotype can generally be detected with three tests. By that criterion we have examined 10.2% of all genes in a putative null state, and 37.7% of all genes in either a putative null or probably damaged state. All this means that we have examined 24% of all genes in the mouse genome, having damaged them sufficiently to detect a phenotype if one could be made through damage, and having checked the mutant alleles three times or more in the homozygous state.

It is possible for us to project how this will go over time. At the present time in the progress of our mutagenesis effort we are right here [22:15]. We have created coding changes in something like 74% of all genes. This is the red curve for probably null or probably damaging; this is the curve for putative null alleles, and this is the curve for mutation to phenovariance—in all cases, with three times examination. We can project how far we will have to go to reach, let’s say, 33% mutation to phenovariance. That should happen sometime around the end of this year, or by the time we have reached a total of approximately 68,000 G3 mice. We can also estimate saturation by the number of mutations that have been screened, or by time (assuming a certain rate of mouse production). All of these curves are predicated on our present rate of screening, which is about 600 mice per week. That means testing about 1,000 mutations per week for phenotypic effects in 150 screens.

In the course of screening, we find things that are known, of course. For the adaptive immune screens alone I made a list this morning. I see that we have found by phenotype a total of 74 genes that were previously known to be needed for adaptive immune function or response, because mutations in these genes created phenotypes in our mice. Of course we are not interested in things that are already known. We are interested in things that are unknown. I estimate that approximately the same number of mutations that have effects that are unknown have also been found and verified. So there is a great deal still to learn about what is needed for the adaptive immune response.

How do we keep track of our mutations and their effects? We developed software that lets us parse the mutations and query them according to gene name or screen name or particular groups of mice. We can also parse according to the predicted effects of mutations, whether they are nonsense or critical splicing errors, or other. We can restrict our search to pedigrees with greater than a certain number of mice. We can restrict our search according to the number of homozygotes for any mutation. We set the *P* value of what we believe is significant and wish to pursue.

I will give you an example from the innate immune screens, where we have recently had some success. Many of you have heard of the NLRP3 inflammasome. NLRP3 is involved in most forms of inflammation—notably in gout, and also in certain diseases such as cold-induced auto-inflammatory disease, or neonatal onset multisystem inflammatory disease (NOMID). It is the protein that organizes the processing of interleukin-1β, an inflammatory cytokine that is secreted and helps to generalize the inflammatory response. We wanted to find all kinds of mutations that affect NLRP3 inflammasome function, either increasing interferon-β production or abrogating it in response to a defined stimulus. Here we focus on that particular screen [25:46]. I won’t go through what trimmed and untrimmed are. We restrict the search to >15 as the total number of mice in the pedigree. We insist on seeing three or more homozygotes for a mutation, and we set the *P* value at 0.005.

When we click [26:08], we find a list of genes. In fact, we retrieve 61 mutations in 60 genes from 46 pedigrees. Some of these will be familiar and some will not. Here is one that is familiar. We mutated NLRP3 itself. In fact, we hit the gene nine times in our collection of mutations, making nine separate mutations. Sometimes one has multiple pedigrees with the very same mutation. Usually that is the result of inheritance from a common G0 progenitor. You see the co-ordinates of the mutation, you see the computer’s declarations about it, that it is a missense allele, probably damaging. You can move over to the right and you see that there are in this pedigree five homozygous wild-types, five heterozygotes, five homozygous mutant mice. By the additive model of inheritance (in other words, semi-dominant model of inheritance), the mutation passes our criterion for the *P* value.

If we want to look at all of the mutations, we can do so by clicking on that value [27:21]. Here is the one of interest. If you mouse over it, you see that this is NLRP3. This is a scale showing the likelihood of association between phenotype and genotype, given random assortment. In other words, there is a likelihood of nearly one in 10^–6^ that the association would occur by chance.

If you did not know anything about this gene, you could click on it again, and the computer has already calculated a lot of information for you with links to the MGI database and other sources of detailed information. It has drawn a gene model and also a protein model. This is the protein model in SMART format [27:59], and it shows that this is a missense mutation of isoleucine to asparagine at position 293. The model is interactive.

You can also look at the gene model by clicking on the link, and you see that the mutation is in exon 5, and not predicted to effect splicing.

As to the phenotypic data, if you left-click on the point [28:26] you see this is the performance of homozygotes, which are poor producers of IL-1β, heterozygotes which are intermediate, and homozygotes for the reference allele. These are all from a common pedigree and can be taken as littermates. It was the allele-additive relationship that made the computer flag the mutation. The wild-type is shown for different purposes, not for comparison.

There are also mutations that would seem new to most of us. Many of you would not guess that a protein called NEK7 is important for the inflammasome response. We have four separate alleles of NEK7. This is a nonsense allele, and we see that the pedigree is quite large. There are a total of 42 G3 mice, and by an additive model the Manhattan plot shows a linkage peak. NEK7 is a member of a kinase family called the NEMA kinases, or the “never in mitosis” kinases. The mutation in question is a premature stop codon. The NEMA kinases are associated with mitosis. They are known to be required for assembly of the spindle apparatus and involved in abscission of cells during late mitosis. Yet nothing was known about their involvement in inflammasome function.

The homozygotes show poor IL-1β responses. Heterozygotes perform better; homozygous wild-type mice perform best of all.

That might not be enough to convince you. Perhaps you are wondering about the other pedigrees we have and what they show. What of the other alleles of NEK7? Fortunately, the computer knows this. It realizes when there are multiple alleles and groups all of the data into superpedigrees. Eventually all of the genes will be included in superpedigrees. At present 61% of all genes have two or more alleles and are in superpedigrees. With multiple alleles one gains confidence about the strength of associations.

Some superpedigrees are spectacular. This is a particular unknown gene that I won’t talk about today. A mutation within it affects the number of CD8 cells. We are looking here at phenotypic performance pooled across about 16 pedigrees. In this case, always the same mutation was involved. This was a case where there was multiple transmission to many G1 mice. Notice there are hundreds of mutations now. Only one of them is above the Bonferroni correction line. That’s the gene in question [31:11].

If you click on it, you can observe the phenotypic performance of this allele in all the different pedigrees, with color coding of each. You see the CD8 count is shifted quite dramatically in homozygotes as compared to heterozygotes or mice with the reference allele. You would not really need to target this gene in view of these data. You could be quite confident that this was the causative mutation. But to guard against the possibility that an unseen mutation might be responsible, we target the gene nonetheless.

The situation isn’t quite so good for NEK7, but NEK7 is above the Bonferroni correction line. Each of three different mutations that reached homozygosity shows a diminished inflammasome response compared to the heterozygote or reference allele populations. So we can feel fairly confident in proceeding and knocking out the gene.

We observed the NEK7 protein in cells from homozygotes for the ENU allele, which we named *Cuties*, is practically gone. The mRNA is eliminated by nonsense-mediated decay, and what little truncated protein is translated is apparently unstable. When we did knock *NEK7* out by CRISPR targeting, we found again that the protein was gone. Furthermore, the NLRP3 inflammasome does not work properly; one can normally activate it with Nigericin, with ATP, or with Alum, all of which are inflammatory stimuli specific for NLRP3. On the other hand, other inflammasomes, such as Nlrc4 which responds to flagellin, or Aim2, which responds to Poly(dA:dT), operate normally in the absence of NEK7—there is no difference in phenotypic performance. We knocked down the gene in human mononuclear cells, and we found that there, too, the inflammasome was suppressed in cells from two different donors.

Notice that IL-18 as well as IL-1β is suppressed in *Cuties* mice or knockout mice. In this case we are looking at another end-point of the NLRP3 inflammasome. We find that cytotoxicity is diminished. This is called pyroptosis, and it is diminished using all of these stimuli. On the other hand, TNF production and IL-6 production are unimpaired in *Cuties* mice.

There are two signals that activate the inflammasome. Signal 1 drives the expression of inflammasome components like NLRP3 itself and also Pro-IL-1β—the target for cleavage by the inflammasome. Signal 2 is generated by reagents like Nigericin or Alum. In a somewhat mysterious way they activate the inflammasome. Signal 1 is unimpaired. Notice that with no treatment little or no NLRP3 or Pro-IL-1β is expressed. With LPS, the first signal, there is a strong response in *Cuties* mice. On the other hand, signal 2 is very much impaired. Homozygotes show almost no response to LPS+Nigericin or LPS+ATP in terms of secreted IL-1β.

Signal 1 is also unimpaired if you look at hallmarks like mitochondrial oxidative radicals or calcium flux into the cell. These responses are essentially the same in *Cuties* and wild-type mice.

We next looked at the assembly of NLRP3 and its association with ASC, a downstream protein that recruits caspase 1, which then cleaves Pro-IL-1β to release IL-1β. This can be followed [35:06] by sucrose density gradient ultracentrifugation. We can see a slight shift of NLRP3 when we do this, toward a heavier weight, if we activate with Nigericin. One can also observe ASC recruitment of the complex, and in *Cuties* mice that is very much impaired compared to wild-type mice. We can also look at the oligomerization of ASC using a cross-linking agent like disuccinimidyl suberate [DSS at 35:36]. In this case you find that with *Cuties* mice that is also very much impaired.

One can see physical association between NEK7 and NLRP3 as well. NEK7 in normal wild-type cells is distributed like this [35:55] in a sucrose gradient, and, on the other hand, NLRP3 is located toward the high-density end of the gradient. When you activate, NEK7 largely overlies the NLRP3 band, indicating that NEK7 may associate with NLRP3 and co-sediment with it.

Looking at how it might interact more directly by expressing tagged versions of NLRP3 and NEK7, we find that the whole protein does associate and co-precipitates. Furthermore, if we break up NLRP3 into its pyrin domain, nucleotide-binding domain, and leucine-rich repeat portions, NEK7 binds to the leucine-rich repeats of NLRP3.

Mutations affecting the leucine-rich repeats of human NLRP3 result in rare cases of NOMID, because they make a constitutively active protein. Two of these are shown here: G775A and G775R. These mutations cause tighter association with NEK7. On the other hand, we have in our collection a mutation in NLRP3 that diminishes the inflammasome response, and here it diminishes the binding of NEK7 to the leucine-rich repeats. This mutation is also in the leucine-rich repeat moiety of NLRP3.

NEK7 is a kinase, but kinase activity is apparently not required for association between NEK7 and NLRP3. Nor is it required for NEK7 to rescue the *Cuties* phenotype. If we take knockout cells and transfect them either with wild-type or with a kinase-dead mutant expression construct, in both cases rescue of the IL-1β non-production phenotype is observed.

Finally, NEK7 is also required for NLRP3 inflammasome activation *in vivo*. *Cuties* mice don’t mount a normal inflammatory response to the intraperitoneal (IP) injection of urate crystals in terms of recruitment of peritoneal exudate cells or neutronphils or macrophage separately. We have also studied experimental allergic encephalomyelitis (EAE) which is an IL-1β-dependent phenomenon, and the NEK7 mutation suppresses that as well. We are intrigued by the fact that NEK7 is a kinase that is known to be involved in mitosis. We blocked cells at different parts of the mitotic cycle and found that during mitosis it was not possible to induce an inflammasome response. This is a measurement of the activation of caspase 1 in mitotic cells compared to interphase cells. We hypothesize that this failure to activate the inflammasome during mitosis reflects the recruitment of NEK7, an essential inflammasome component, to fulfill duties required elsewhere in the cell during mitosis. The evolutionary explanation for this recruitment might be that it prevents damage to DNA that would occur during inflammasome activation, at a time when chromosomes are condensed and when repair can’t take place as it ordinarily would.

We now view the inflammasome quite differently than before. We would say that in response to signal 2 there might be activation of the NLRP3 molecule itself, and there are a number of proposed mechanisms for that. But it could just as well be that activating signals impinge upon NEK7. Whatever the activation mechanism, it is clear that NEK7 binds to the leucine-rich repeats of NLRP3 and that is necessary for activation of caspase 1 and the generation of IL-1β, IL-18, IL-33, and all that follows downstream in the inflammatory cascade. Nobody has ever managed to crystallize the NLRP3 inflammasome; that may be because they have been missing this important component that affects the solubility of the molecule. We are attempting to do structural studies now on the complex between NEK7 and NLRP3.

## SUMMARY

We have damaged or destroyed about one-quarter of all genes in the mouse, testing the mutant alleles three times or more in the homozygous state, focusing on the effects of these mutations on immune function and some neurobehavioral functions. As phenotypes are seen, we are able to link cause and effect quite reliably and very quickly: within about an hour. Speaking as one who lived through the era of five-year positional cloning projects, that is amazing.

We have identified many new proteins required for normal biological functions. We can even make some preliminary estimates of how many essential proteins there are for one function or another, based on the fact that we have gone through a quarter of the genome and assuming that size is independent of criticality.

We can predict that we will reach half saturation for some of our screens within about three years at the present rate of production and testing of mice.

In the specific example I showed you, we found a new component of the NLRP3 inflammasome, something important all by itself, but it was one of many stories still in progress. The solution of mechanisms remains the limiting factor at this point.

It takes a large team of scientists to automate genetic mapping and solve phenotypes instantly. You see, I am going against my father’s advice about the size of one’s lab versus the size of one’s ideas. But in fact, it takes quite a number of people to maintain the pipeline to discover new biological functions, just as I have shown you in this process. The person who deserves the most credit for the NLRP3 story is Hexin Shi, but no doubt he had help from everyone shown here. They all work together co-operatively.

Thanks again for the privilege of talking to you and seeing your work unfold.

## 

## Abbreviations

CRISPR: clustered regularly interspaced short palindromic repeats
ICSI: intracytoplasmic sperm injection
IP: intraperitoneal
IVF: *in-vitro* fertilization
NOMID: neonatal onset multisystem inflammatory disease

